# Empiric treatment of protracted idiopathic purpura fulminans in an infant: a case report and review of the literature

**DOI:** 10.1186/1752-1947-5-201

**Published:** 2011-05-23

**Authors:** Fima Macheret, Kavitha N Pundi, Eileen M Broomall, Dawn M Davis, Vilmarie Rodriguez, Chad K Brands

**Affiliations:** 1 Mayo Medical School, 200 First Street SW, Rochester, MN 55905, USA; 2 Department of Pediatric and Adolescent Medicine, 200 First Street SW, Rochester, MN 55905, USA; 3 Department of Pediatric Dermatology, 200 First Street SW, Rochester, MN 55905, USA

## Abstract

**Introduction:**

Idiopathic purpura fulminans is a cutaneous thrombotic disorder usually caused by autoimmune-mediated protein C or S deficiency. This disorder typically presents with purpura and petechiae that eventually slowly or rapidly coalesce into extensive, necrotic eschars on the extremities. We present the first known case of idiopathic purpura fulminans consistent with prior clinical presentations in the setting of a prothrombotic genetic mutation, but without hallmark biochemical evidence of protein C or protein S deficiency. Another novel feature of our patient's presentation is that discontinuation of anti-coagulation has invariably led to recurrence and formation of new lesions, which is unexpected in idiopathic purpura fulminans because clearance of autoimmune factors should be followed by restoration of anti-coagulant function. Although this disease is rare, infants with suspected idiopathic purpura fulminans should be rapidly diagnosed and immediately anti-coagulated to prevent adverse catastrophic outcomes such as amputation and significant developmental delay.

**Case presentation:**

A six-month-old Caucasian boy was brought to our pediatric hospital service with a low-grade fever and subacute, symmetric, serpiginous, stellate, necrotic eschars on his forearms, legs and feet that eventually spread non-contiguously to his toes, thighs and buttocks. In contrast to his impressive clinical presentation, his serologic evaluation was normal, and he was not responsive to corticosteroids and antibiotics. Full-thickness skin biopsies revealed dermal vessel thrombosis, leading to a diagnosis of idiopathic purpura fulminans and successful treatment with low-molecular-weight heparin, which was transitioned to warfarin. Long-term management has included chronic anti-coagulation because of recurrence of lesions with discontinuation of treatment.

**Conclusion:**

In infants with necrotic eschars, it is important to first consider infectious, inflammatory and hematologic etiologies. In the absence of etiology for protracted idiopathic purpura fulminans, management should include tissue biopsy, in which thrombotic findings warrant a trial of empiric anti-coagulation. Some infants, including our patient, may need long-term anti-coagulation, especially when the underlying etiology of coagulation remains unidentified and symptoms recur when treatment is halted. Given that our patient still requires anti-coagulation, he may have a yet to be identified autoimmune-mediated mechanism for his truly idiopathic case of protracted purpura fulminans.

## Introduction

Purpura fulminans (PF) is caused by a relative deficiency in protein C-mediated hemostasis and is classified by etiology and history into neonatal PF, acute infectious PF and idiopathic or post-infectious PF. Neonatal PF is caused by homozygous or compound heterozygous deficiencies in protein C or protein S and manifests shortly after birth [1]. In contrast, acute infectious PF occurs at any age and is most likely a consequence of coagulative consumption of protein C, protein S and anti-thrombin III during sepsis [2,3]. It is considered a dermatologic manifestation of endotoxin-triggered hemostatic abnormalities (specifically, induced protein C and S deficiency) associated with sepsis and disseminated intravascular coagulation (DIC), most frequently in the setting of serogroup C meningococcemia and endotoxemia [4,5].

Idiopathic PF, an extremely rare disease, is attributed to the development of anti-protein S antibodies, which form antibody-protein S complexes that are excreted, leading to transient protein S deficiency, hypoactivation of the protein C pathway and dermal vessel hypercoagulability. Important evidence suggests a post-infectious etiology. After D'Angelo *et al. *[6] identified autoantibodies to protein S in a boy with thromboembolic disease antecedent to varicella infection, Levin *et al. *[7] discovered and quantified protein S autoantibodies in four children with idiopathic PF following varicella infection. Indeed, Regnault *et al. *[8] confirmed that protein S autoantibodies obtained from a child with idiopathic PF antecedent to varicella infection caused a hypercoagulable state *in vitro*. All of the patients in the Levin *et al. *[7] case series had minimal or undetectable protein S and protein S activity. Some of the patients in the Levin *et al. *series had other laboratory abnormalities, including elevated anti-cardiolipin immunoglobulin M (IgM) and IgG antibodies, IgG-containing immune complexes, decreased C4B binding protein and decreased protein C activity.

Despite the important work by D'Angelo *et al. *[6] and Levin *et al. *[7] with regard to post-varicella etiology, there may be other anti-coagulant factors involved in the disease process. The independent contribution of heterozygous prothrombin G20210 mutations to the development of autoimmune protein S deficiency and idiopathic PF is still unclear. This mutation has been reported in two cases of idiopathic PF and autoantibodies to protein S with decreased protein S levels [9,10]. The presence of anti-phospholipid antibodies has also been reported in conjunction with protein S deficiency and thrombosis in children [11]. These cases underscore that work-up for suspected idiopathic PF should include clotting factor assessment, rheumatologic evaluation and thrombophilic mutation analysis.

## Case presentation

A previously healthy six-month-old Caucasian boy was brought to our pediatric hospital service for a second opinion regarding a six-week history of necrotic skin lesions on his upper and lower extremities. The lesions were initially petechial and rapidly evolved into escharotic plaques over a 10-day period. They were accompanied by the onset of a low-grade intermittent fever and night sweats. One month after his initial presentation, the lesions had spread to involve the distal, dorsal surface of his feet and toes. His parents denied any trauma, burns or other inciting injuries.

He had an extended family history of stroke, miscarriages, deep venous thromboses, pulmonary emboli and multiple sclerosis, but his parents denied any coagulopathies, bleeding disorders or rheumatologic diseases. His social history was non-contributory. His vaccinations were up-to-date, and he had received his most recent ones about three weeks before the onset of the lesions, which included vaccinations for diphtheria-tetanus-acellular pertussis, haemophilus influenzae type B, inactivated polio vaccine, hepatitis B virus, heptavalent pneumococcal vaccine and rotavirus vaccine.

Outside investigations included a negative skeletal survey and normal laboratory studies. He received a short, unsuccessful course of methylprednisolone. At the time of presentation, his only other medications were ranitidine (One and a half mg/kg/day) and omeprazole (0.67 mg/kg/day) for gastroesophageal reflux disease and lactulose for a recent history of constipation that began after rice cereal was added to his diet.

**Figure 1:**
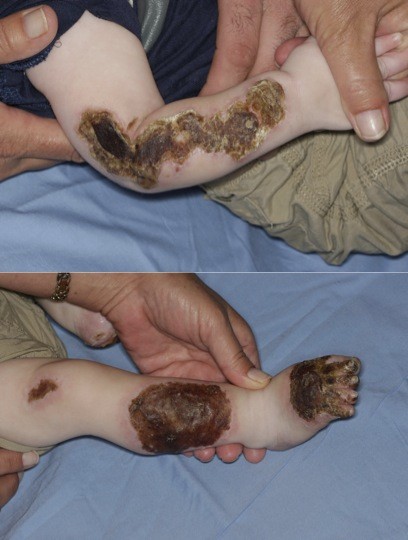
**Necrotic skin lesions at time of first admission**. Right forearm is shown in the top panel, and right leg and foot are displayed in the bottom panel.

Upon admission to our hospital, his physical examination revealed an infant who was alert, active and intermittently fussy. His vital signs were heart rate 128 beats/minute, blood pressure 126/94 mmHg, respiratory rate 42 breaths/minute and body temperature 36.9°C. His skin examination revealed blackened, eschar-like, linear but serpiginous, stellate, spatially non-contiguous lesions with peripheral yellow crusting. There were symmetrical necrotic plaques on his forearms, distal legs, dorsal surface of his feet and his toes, including the first two toes on each foot (Figures 1a and 1b). Otherwise his physical examination was non-contributory.

The results of our initial laboratory investigation are shown in Table 1. Additionally, a peripheral smear revealed Döhle bodies with toxic granulations. Serum protein electrophoresis revealed hypogammaglobulinemia (γ-globulins 0.3 g/dL). Serial blood cultures were negative for bacteria and fungi.

**Table 1:** Laboratory evaluation at first admission, dismissal and second admission^a^

Test or laboratory evaluation	Admission 1 (dismissal 1)^b^	Admission 2	Reference range
Hemoglobin	13.1 (9.6^c^)g/dL	11.5 g/dL	10.5 to 13.5 g/dL
Hematocrit	37.5 (27.6^c^)%	34.0%	33.0% to 44.0%
Leukocytes	16.5^c ^(15.7^c^) × 10^9^/L	26.3 × 10^9^/L^c^	6 to 11 × 10^9^/L
Thrombocytes	738^c ^(651^c^) × 10^9^/L	951 × 10^9^/L^c^	150 to 450 × 10^9^/L
Neutrophils	11.41^c ^(6.47) × 10^9^/L	-	1.5 to 8.5 × 10^9^/L
Lymphocytes	3.91^c ^(7.65) × 10^9^/L	-	4.0 to 10.5 × 10^9^/L
Monocytes	1.09 (1.42^c^) × 10^9^/L	-	0.05 to 1.1 × 10^9^/L
Eosinophils	0.00^c ^(0.05) × 10^9^/L	-	0.05 to 0.7 × 10^9^/L
PT	9.8 seconds	9.6 seconds	8.3 to 10.8 seconds
aPTT	30 seconds	30 seconds	21 to 33 seconds
Thrombin time	20 seconds	22 seconds	16 to 25 seconds
Protein C, activity	99%	120%	70% to 150%, adults
Protein S, free	116	144	65% to 160%, men
Protein S, activity	123%	123%	65% to 160%, adults
Anti-thrombin activity	119%	136%^c^	80% to 130%, > 6 months old
D-dimer		200 ng/mL	< 250 ng/mL
Fibrinogen	315 mg/dL	351 mg/dL	200 to 375 mg/dL
DRVVT		1.0	< 1.2
APC ratio		3.1	> 2.3
Factor VIII		170%	55% to 205%, adults
Ristocetin co-factor		92%	55% to 200%, adults
von Willebrand factor		112%	-
ESR	11 mm/hour	25 mm/hour	-
CRP	5.6 mg/L	9.1 mg/L^c^	< 8.0 mg/L
ANA antibody	0.1 U		< 1.0
Homocysteine	7 μmol/L	5 μmol/L	< 13 μmol/L
Anti-phospholipid/anti-cardiolipin, IgM	< 4.0		-
-IgG	< 4.0		-
AST	27 U/L^c^	24 U/L	8 to 20 U/L
ALT	18 U/L	15 U/L	8 to 20 U/L
ACE	16 U/L		8 to 53 U/L
Cryofibrinogen	Neg		Neg
Cryoglobulin	Neg		Neg
Plasma porphyrins	< 1 U/L		< 1 U/L
RBC ALA dehydratase	8.7 nmol/L/second		-
Myeloperoxidase	< 0.2 U	< 0.2 U	< 0.4 U
Proteinase 3	< 0.2 U	< 0.2 U	< 0.4 U
Total protein	5.3 g/dL^c^		6.0 to 7.8 mg/dL
Albumin	3.0 g/dL^c^		3.5 to 5.5 mg/dL
Sodium ion		138 mmol/L	135 to 145 mmol/L
Potassium ion		5.2 mmol/L^c^	3.5 to 5.0 mmol/L
Bicarbonate ion		19 mmol/L	18 to 23 mmol/L
Chloride		105 mmol/L	95 to 105 mmol/L
BUN		9 mg/dL^c^	3 to 7 mmol/L
Creatinine		0.2 mg/dL^c^	0.7 to 1.0 mg/dL
GGT		19 U/L	5 to 40 U/L
Anion gap		14 mEq/L^c^	8 to 12 mEq/L
Glucose		107 mg/dL^c^	70 to 99 mg/dL
Bilirubin (total/direct)		0.2/0.1 mg/dL	0.1 to 1.0/0.0 to 0.3 mg/dL
IgM	80 mg/dL	67 mg/dL	24 to 267 mg/dL
IgG	272 mg/dL	289 mg/dL	164 to 588 mg/dL
IgA		130 mg/dL^c^	16 to 50 mg/dL
IgE		7.5 kU/L	< 30 kU/L
Complement C3		128 mg/dL	75 to 175 mg/dL
-C4		134 mg/dL^c^	14 to 40 mg/dL
Fc C7		59 U/mL	36 to 60 U/mL
-C8		60 U/mL^c^	33 to 58 U/mL
-C9		61 U/mL	37 to 61 U/mL
β_2 _microglobulin		5.6 μg/mL	0.70 to 1.80 μg/mL
Zinc		0.67 μg/mL	0.6 to 1.20 μg/mL
α_1_-anti-trypsin		144 mg/dL	89 to 230 mg/dL
Cold agglutinin	(Negative)		
Mycoplasma IgM	0.10		< 0.90
-IgG	0.5		-

^a^PT, prothrombin time; aPTT, activated partial thromboplastin time; DRVVT, dilute Russell's viper venom test; APC, activated protein C; ESR, erythrocyte sedimentation rate; CRP, C-reactive protein; ANA, anti-nuclear; IgM, immunoglobulin M; AST, aspartate aminotransferase; ALT, alanine aminotransferase; ACE, angiotensin-converting enzyme; RBC ALA, red blood cell Δ-aminolevulinic acid; BUN, blood urea nitrogen; GGT, γ-glutamyl transferase; Fc, functional component; ^b^values in parentheses are those which occurred at first discharge, which was approximately one week after first admission. The second admission occurred 1 week after the first discharge. ^c^outside normal limits.

Punch biopsies from all four limbs revealed a pauci-inflammatory occlusive vasculopathy. Direct immunofluorescence showed strong IgM staining in the superficial and mid-dermal vessels, weak C3 staining in a few superficial vessels and some fibrinogen deposition in the superficial and mid-dermal vessels. Staining for IgG and IgA was negative. Excisional biopsy tissue was cultured and revealed anaerobic Gram-positive cocci (3+), enterococci (3+), *Neisseria animaloris *(2+) and a few white blood cells, and stains of the biopsy were negative for acid-fast organisms, mycobacterium, and fungi. Immunofluorescence staining was negative for IgG, C3 and fibrinogen, while IgA stained weakly positive in rare dermal blood vessels and IgM stained positive in a few scattered blood vessels in the dermis.

The patient was discharged on wet-to-dry dressing changes, and his laboratory abnormalities at dismissal are shown in Table 1. Prednisolone therapy was tapered over two weeks.

The patient was re-admitted one week later because of progression of his skin disease. Flexion contractures were noted at the elbows and knees. Laboratory values at readmission are shown in Table 1. Genetic analysis revealed that he was a heterozygous carrier of the prothrombin 20210A mutation. Further imaging included computed tomography of the chest and abdomen, which were unrevealing. Additional viral and retroviral studies were all negative, including varicella. His oxidative burst test was also normal. CD11a was not indicative of leukocyte adhesion deficiency. Tests for arsenic, lead, mercury and cadmium were all negative.

## Discussion

After the extensive negative work-up and given the lack of a definitive diagnosis, a peripheral inserted central catheter line was placed for the initiation of high-dose IV prednisone (Four mg/kg/day). He was started empirically on IV ampicillin/sulbactam (200 mg/kg/day, seven-day course). Corticosteroids were tapered over the next seven days.

One week later he showed no clinical improvement. Following interdisciplinary conference and consultation with pediatric coagulation experts, we decided to treat him empirically for idiopathic purpura fulminans with dalteparin (two SQ doses at 250 U/kg/day). This regimen was adjusted to 350 U/kg/day divided into two SQ doses to achieve a therapeutic heparin level. Acetylsalicylic acid was also added. Physical therapists were consulted because of motor skill regression with skin contractures.

**Figure 2:**
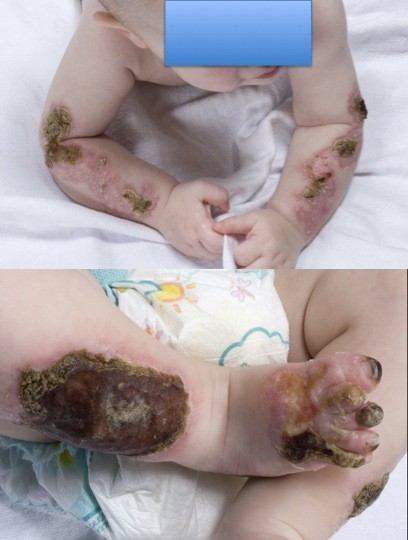
**Healing skin lesions one week after initiation of anti-coagulation**. Both arms are shown in the top panel. Right leg and right foot are displayed in the bottom panel. No new lesions had formed, and pink, healing scars had begun to form under the eschars.

At his follow-up examination one week later, his parents reported that no new lesions had developed. The eschars had begun to regress centrifugally from their borders, revealing healing pink scars, especially in his upper extremities. After several weeks, peripheral areas of crusting had shed, revealing healed, scarred skin (Figures 2a and 2b).

**Figure 3:**
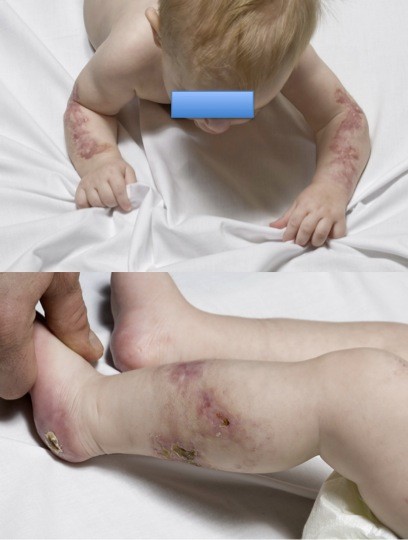
**Healing skin lesions two months after initiation of therapy**. Both arms are shown in the top panel. Left leg and left foot are shown in the bottom panel. Eschars have completely given way to pink, healing scars with no new lesions formed.

Follow-up at two months demonstrated dramatic healing (Figures 3a and 3b). While on heparin therapy, he had transient recurrence of petechiae one day after receiving an H1N1 virus vaccination. Any attempt to reduce the low-molecular-weight heparin from twice to once daily led to symmetric recurrence of lesions on his buttocks and thighs. He was transitioned to chronic anti-coagulation with warfarin 1 mg/day, and the lesions have been well controlled when his international normalized ration is in the therapeutic range of two to three. Subsequent urine amino acid analysis and genomic microarray analyses were unremarkable.

About one year after initial presentation, our patient was experiencing developmental delays that were especially noted in gross motor function. Neurology consultation was sought, and their work-up revealed proximal muscle weakness and areflexia. Further investigation revealed that he is heterozygous for a duplication of a thymine nucleoside in his *SMN1 *gene at position 91 in codon 31, which causes a frame shift mutation and is consistent with spinal muscular atrophy.

The differential diagnosis for an infant with necrotic skin lesions is listed in Table 2. After interdisciplinary consultation, we arrived at a diagnosis of idiopathic PF, a rare prothrombotic syndrome characterized by thrombosis of dermal vasculature and progressive, widespread purpura with necrosis. Our patient's extensive characteristic lesions and stereotypical features on skin biopsy were consistent with those described in prior reported cases. Indeed, our patient's impressive response to anti-coagulation was strikingly similar to that described in prior cases and argues in favor of this diagnosis.

**Table 2:** Differential diagnosis of necrotic skin lesions in an infant^a^

System	Diagnoses
Hematologic	Idiopathic thrombocytopenic purpura Thrombotic thrombocytopenic purpura Coagulation factor deficiency Platelet function disorder Thromboembolic disease Lupus/anti-phospholipid antibodies Cryoglobulinemia Porphyria Idiopathic red blood cell agglutination Idiopathic PF Neonatal PF or other thrombophilic mutation
Infectious	Cellulitis with or without atypical organisms or fungi Endocarditis (bacterial, fungal or marantic)
Inflammatory	Panniculitis secondary to α_1_-anti-trypsin deficiency Pemphigus vulgaris Pyoderma gangrenosum Epidermolysis
Vascular	HSP Leukocytoclastic vasculitis Hematoma with compartment syndrome
Neoplastic	Leukemia Lymphoma Essential thrombocythemia Cutaneous T-cell lymphoma
Nutritional	Vitamin deficiency (vitamin K) Mineral deficiency (zinc)
Environmental	Toxic exposure (for example, heavy metals)
Congenital	Combined immune deficiency Prolidase deficiency Leukocyte adhesion deficiency Chronic granulomatous disease γ-globulinemia Spinal muscular atrophy
Trauma	

^a^PF, purpura fulminans; HSP, Henoch-Schonlein purpura.

The most novel divergence of our patient's idiopathic PF from prior cases was the disparity in laboratory evidence. Fascinatingly, our patient had no absolute or relative deficiencies in protein C and S levels or activities, features present in the prior reported cases. Protein C activity was measured using venom activator and chromogenic peptide substrate; therefore, it is possible that a congenital protein C variant may not have been identified that would require a clot-based assay. Protein S antigen was measured using free protein S assay, and protein S activity was measured using a clot-based end point. This protein S assay can be affected by the presence of lupus anti-coagulant or a specific factor V inhibitor, but we did not identify either of these humoral factors.

Also, the peripheral smear abnormalities, especially the Döhle bodies, can be associated with DIC-linked purpura fulminans, yet we saw no evidence of consumptive coagulopathy characteristic of DIC. The specific role of *N. animaloris *in the progression of his lesions remains undefined. He may have a novel form of idiopathic PF, possibly preceded by an unidentified infection or caused by a defect in protein S that we were unable to detect using conventional laboratory assays. We are also unable to assess the potential contribution of his series of vaccines to his disease presentation, and although it would be unlikely for a child to develop autoantibodies before 6 months of age, we do not rule out the possibility that he may have developed autoantibodies after immunization.

Another important aberration in the patient's presentation was his age, because he was older than the expected age for neonatal PF and younger than all of the other reported cases of idiopathic PF that we reviewed. While our patient's initial response to anti-coagulation therapy was the aspect of his presentation and outcome, which fit most closely with that of idiopathic PF, his long-term need for anti-coagulation is not consistent with the previously described etiology of this disease.

The photographs associated with this case offer a dramatic example of clinical management for the pediatrician or dermatologist who may see similar lesions in a non-septic infant with an unrevealing laboratory evaluation. The decision to empirically anti-coagulate represented our best scientific response to the overlapping and non-specific findings for idiopathic PF.

In light of his impressive response to treatment as depicted in the final set of images (Figure 3), we wish to highlight an evidence-based approach to empiric treatment of idiopathic PF. Table 3 summarizes the patient age, gender, protein S levels, varicella status, treatments and outcomes of several recent cases reported after the Francis case series [12]. On the basis of the hypothesized etiology of the disease, that is, the development of protein S autoantibodies with thrombogenesis, treatment should include plasmapheresis with intravenous immunoglobulin to eliminate protein S autoantibodies and heparin to improve hemostasis [13]. Fresh frozen plasma should be added to assist the recovery of clotting factors. The dosage of dalteparin was titrated up to 350 U/kg twice daily to reach a target anti-Xa level of 0.5 to 1.0. We also prescribed supplemental 81 mg aspirin once daily. Other emerging modalities have been used to treat acute infectious and neonatal PF and may be applicable in some cases of idiopathic PF, including protein C replacement, anti-thrombin III replacement, prostacyclin and even leech therapy [3,14]. Long-term management must include physical therapy to assist with weight-bearing if there is any evidence of contracture.

**Table 3:** Treatment modalities for idiopathic purpura fulminans from recently reported cases^a^

Case report	Patient and history	PS	Trigger	Treatment (target levels)^b^	Outcome and treatment^c^
Boccara *et al. *[15]	Two-year-old girl Varicella three months prior	Low	HHV-6	IVIg 2 g/kg, LMWH, plasmapheresis q12 h, FFP (repeat IVIg if PS < 50%)	Pre-treatment: right leg amputation Post-treatment: resolution, IVIg 2 ×/day
Özbek *et al. *[9]	Eight-year-old boy FVL^+/-^ G20210A^+/-^	Low	Not known	Prior treatment: SQ UH, IV ceftriaxone, metronidazole (One) Bolus 75U UH, 15 days continuous 20 U/kg/hour, twice daily FFP × 5 days^b^ (Two) 80 to 100 U/kg SQ heparin for 1 month (Three) Warfarin (INR Two or Three)	Pre-treatment: necrosis in lower half of body Post-treatment: resolution
al-Ismail *et al. *[10]	4.8-year-old boy G20120A^+/-^ ANA^+^ ASO^+^ Viral titer^-^	Low	Streptococcus?	(One) Antibiotics, FFP, vitamin K (Two) IV UH (aPTT 1.5-2.5 × normal)^b^ (Three) Enoxaparin 20 mg/q12 hours (anti-Xa level 0.6 U/L)	Pre-treatment: left leg swelling, edema, left femoral vein thrombosis Post-treatment: resolution
Levin *et al*.^d ^[7]	6.4-year-old boy Varicella One year prior	Low	Fever, vesicular rash	(One) IV penicillin, cefotaxime, 4U FFP, IVIg 1 g/kg, exchange transfusion, methylprednisone, IV heparin 15 U/kg/hour, tPa for PE, prostacyclin (Two) IV heparin increased to 60 U/kg/hour, FFP 40 mL/kg/day (aPTT 1.5 to 2.5 × normal)^b^ (Three) Warfarin	Pre-treatment: lesions stopped, right leg amputation, PE^1^ Post-treatment: then respiratory improvement, resolution
	5.9-year-old girl	Low	Varicella	(One) IV cefotaxime, acyclovir and heparin bolus 100 U/kg/hour, then 25 U/kg/hour; two volume exchange transfusion, then daily FFP	Pre-treatment: right atrial thrombosis, PE^e^ Post-treatment: skin grafting

^a^PS, protein S; INR, international normalized ratio; HHV-6, human herpesvirus 6; IVIg, intravenous immunoglobulin; LMWH, low-molecular-weight heparin; FFP, fresh frozen plasma; PS, protein S; FVL, factor V Leiden; G20210A, prothrombin G20210A mutation; SQ, subcutaneous; UH, unfractionated heparin; IV, intravenous; ANA, anti-nuclear antibody; ASO, anti-streptolysin O antibody; aPTT, activated partial thromboplastin time; tPa, tissue plasminogen activator; PE, pulmonary embolism; +/-, heterozygote; +, antibodies present; ^b^most effective treatment modality in each case when more than one treatment was applied; ^c^pre- and post-treatment are with respect to most effective treatment in "Treatment (target levels)" column; ^d^Levin *et al. *[7] case series comprised five cases, but only two had sufficient clinical information both of which are shown here; ^e^both PEs occurred after initiation of effective treatment but before resolution, which occurred after indicated treatments.

Responses to treatment in the referenced cases were similar to those of our patient. Lesions regressed as early as one day after therapy was initiated. Protein C and protein S levels often took weeks to months to return to normal reference levels. As noted in Table 3 some patients lost extremities to gangrene, emphasizing the need for immediate initiation of anti-coagulation as well as surgical excision of gangrene to prevent subsequent sepsis.

## Conclusion

We report the first known case of idiopathic PF without protein C or S deficiency and necessitating chronic anti-coagulation. Pediatric patients presenting with indolent, symmetric, necrotic, eschar-like lesions who have an unrevealing infectious, inflammatory and hematologic work-up should undergo an empiric trial of anti-coagulation to help prevent downstream catastrophic consequences of PF. Starting anti-coagulation decisively may also help to prevent developmental delays in rapidly growing infants with this disease.

## Consent

Written informed consent was obtained from the patient's next-of-kin for publication of this case report and any accompanying images. A copy of the written consent is available for review by the Editor-in-Chief of this journal.

## Abbreviations

ACE: angiotensin-converting enzyme; ANA: anti-nuclear antibody; aPTT: activated partial thromboplastin time; ASD: atrial septal defect; AST/ALT: alanine/aspartate transaminase; AT III: anti-thrombin III; BUN: blood urea nitrogen; Cr: creatinine; CRP: C-reactive protein; DRVVT: dilute Russell's viper venom test; ECG: electrocardiogram; ESR: erythrocyte sedimentation rate; HSP: Henoch-Schonlein purpura; PDA: patent ductus arteriosus; PO: *per os *(by mouth); PT: prothrombin; RBC ALA: red blood cell Δ-aminolevulinic acid; SQ: subcutaneous.

## Competing interests

The authors declare that they have no competing interests.

## Authors' contributions

FM, KP, EB and CB admitted the patient to the pediatric hospital service, performed the patient work-up and wrote the case report. FM was a major contributor in writing the manuscript and did the literature review. DD and VR conducted additional dermatologic and hematologic investigations, respectively. All authors read and approved the final manuscript.
